# An *in-silico* comparative cross-sectional diagnostic- accuracy study evaluating artificial intelligence platforms in identifying autism spectrum disorder from standardized pediatric case vignettes 2026

**DOI:** 10.3389/fpsyt.2026.1941175

**Published:** 2026-09-15

**Authors:** Suhail H. Serbaya, Saud Hasan Surbaya, Tamara Abdulrahman Hafiz, Mohammad Fouad Mohammad Khatib Sambas, Khalid Mohammed Saeed Alshahrani

**Affiliations:** 1 Department of Industrial Engineering, Faculty of Engineering, King Abdulaziz University, Jeddah, Saudi Arabia; 2 Primary Health Care Executive Administration, Makkah Healthcare Cluster, Makkah, Saudi Arabia; 3 Health Promotion and Health Education, Public Health Field, Makkah, Saudi Arabia; 4 Department of Infectious Diseases, King Abdulaziz Hospital, Makkah Healthcare Cluster, Makkah, Saudi Arabia; 5 Alaziziah Primary Health Care Center, Makkah Healthcare Cluster, Makkah, Saudi Arabia

**Keywords:** artificial intelligence (AI), autism spectrum disorder (ASD), classification performance, concordance, diagnostic accuracy, *in silico* evaluation, pediatric case studies

## Abstract

**Background and objective:**

This study evaluates the capability of various general-purpose and healthcare-specialized Artificial Intelligence (AI) platforms in identifying Autism Spectrum Disorder (ASD) from clinical narratives.

**Methods:**

Using twenty standardized pediatric case reports (10 ASD and 10 non-ASD), the evaluation assessed diagnostic accuracy, concordance with clinical diagnoses, and statistical performance across different AI architectures.

**Results:**

The platforms demonstrated diverse operational profiles; Gemini 3 Pro achieved the highest rates of sensitivity and specificity, while other evaluated models exhibited sensitivity rates ranging from 60% to 90%. While statistical differences in performance between general-purpose and specialized systems were not significant (P> 0.799), advanced large language models showed the ability to reason through complex diagnostic narratives.

**Conclusion:**

These findings suggest that advanced general-purpose AI platforms can offer valuable support in interpreting complex ASD case narratives. To ensure clinical safety, incorporating stratified frameworks and standardized protocols remains essential. Further evaluation is required to determine the precise role of these tools.

## Graphical Abstract

**Figure:**
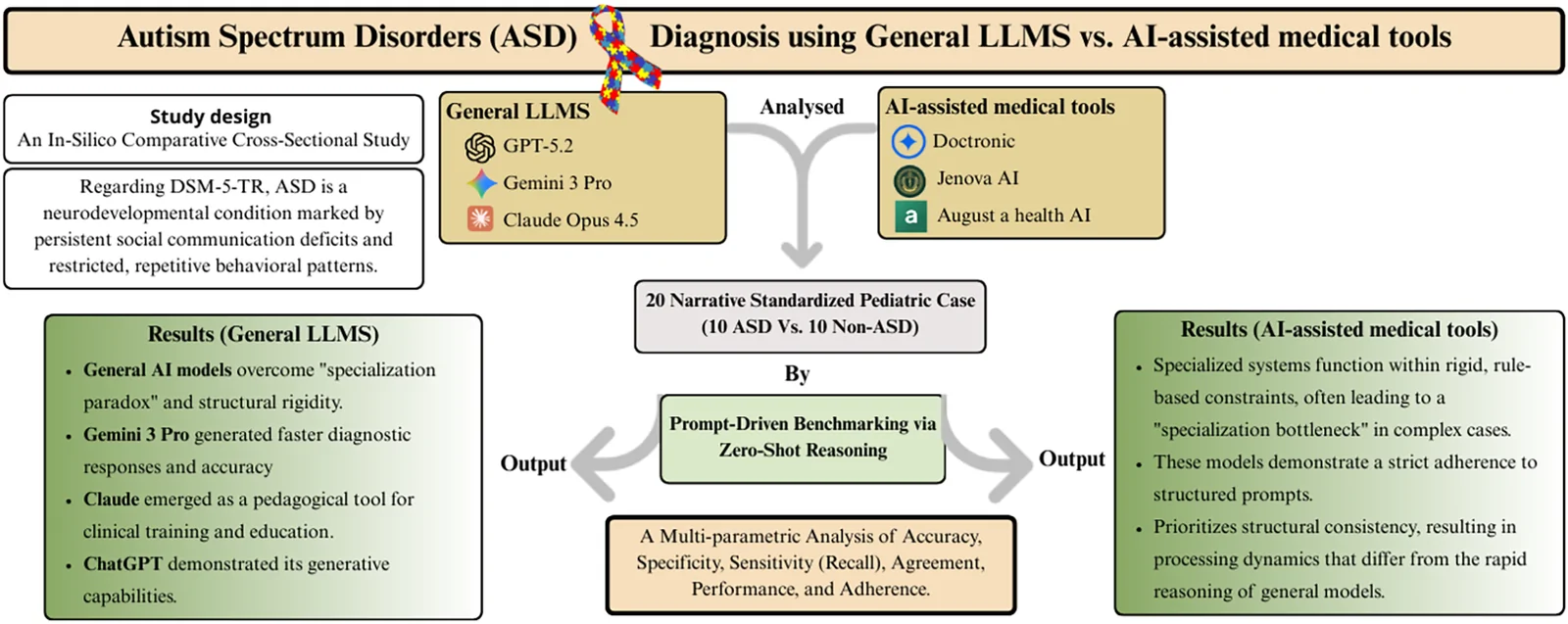

## 1 Introduction

Artificial intelligence (AI) has rapidly evolved, becoming embedded across diverse professional domains. Continuous advancements have enhanced its computational capabilities and potential to address complex, multifaceted tasks. Notably, agentic AI enables machine learning systems and LLMs to autonomously perform complex tasks via external tools and APIs without continuous human supervision. By analyzing large-scale, high-dimensional datasets in real time, AI represents a paradigm shift, particularly within healthcare (1).

Contemporary healthcare systems face mounting pressures, including escalating patient volumes, constrained resources, an increasing prevalence of chronic conditions, and operational time limitations that can impede comprehensive diagnosis and management in hospitals, clinics, and other healthcare facilities. Within this context, AI offers transformative opportunities, enabling clinicians and healthcare providers to systematically interpret patient data, discern intricate clinical patterns, and generate structured insights that facilitate comprehensive reasoning, optimize workflow efficiency, and support evidence-based decision-making (2). Moreover, AI-driven predictive analytics hold the promise to redefine disease surveillance, outbreak preparedness, and clinical decision support, ultimately enhancing the precision, timeliness, and quality of patient care (3, 4). In parallel, recent research has explored AI performance in real-world clinical tasks. Studies using ChatGPT (OpenAI, CA, USA) reveal that while AI-generated documentation eases provider workload and is viewed positively, concerns persist regarding output reliability and validity. These findings emphasize the need for cautious AI integration into clinical workflows (5).

AI’s role is critical in medical specialties requiring nuanced interpretation and high precision, such as pediatric psychiatry. In this field, early, accurate diagnosis profoundly impacts a child’s developmental trajectory and long-term outcomes. Autism spectrum disorder (ASD) is a complex, heterogeneous condition characterized by diverse behavioral, social, and cognitive patterns. Due to its multifaceted clinical presentation, ASD requires the comprehensive assessment and integrative analysis evaluated in this study (6). Many parents report utilizing non-medical interventions as initial approaches, followed by biomedical and cultural or religious therapies. Diagnosing ASD is inherently challenging, requiring clinicians to integrate observations across multiple domains of functioning and incorporate collateral information from caregivers, teachers, and other informants. The severity of ASD varies widely, ranging from mild to profound cases, with severe presentations demanding substantial and ongoing support. Individuals with ASD frequently exhibit repetitive behaviors and demonstrate resistance to changes in daily routines (6, 7). As a result, clinicians must navigate numerous clinical questions and hypotheses to establish an accurate diagnosis—making the process time-intensive and cognitively demanding.

ASD has shown a significant longitudinal increase in prevalence, with U.S. CDC surveillance data reporting a rise from 1 in 59 children in 2006 to 1 in 36 by 2020, highlighting its status as a leading neurodevelopmental priority (8). The rise in ASD prevalence over the past two decades underscores its growing public health significance (6). However, diagnosis remains challenging due to the absence of direct laboratory tests or specific biomarkers; instead, it relies on systematic behavioral assessment and developmental evaluation (9). The fifth edition of the Diagnostic and Statistical Manual of Mental Disorders (DSM-5), published in 2013, revised the diagnostic criteria by consolidating previous subcategories under a single spectrum, such as Pervasive Developmental Disorders (PDD), Autistic Disorder, and Asperger’s syndrome into a single spectrum (10). Furthermore, ASD often co-occurs with other conditions such as attention-deficit/hyperactivity disorder (ADHD), social anxiety disorder, depression, and intellectual disability (11, 12).

A range of screening and diagnostic tools exist to identify ASD. Commonly used screening instruments include the Modified Checklist for Autism in Toddlers (M-CHAT), while less frequently used tools such as the Communication and Symbolic Behavior Scales (CSBS) may also assist early detection (13). For diagnostic confirmation, the Autism Diagnostic Observation Schedule, Second Edition (ADOS-2), remains the “gold standard” (14), offering a semi-structured, standardized method for evaluating social interaction and play across age groups (15). Despite these tools, many children receive diagnoses between 18 and 36 months of age, delaying timely interventions (16). Earlier detection is often associated with more severe symptomatology. Although no cure exists, early diagnosis and intervention enable children to acquire essential developmental, communication, social, and motor skills (17). Additionally, early intervention reduces the substantial economic, emotional, and educational burdens experienced by families and healthcare systems (18, 19).

Mirroring international trends, a recent systematic review in Saudi Arabia analyzed 11 studies (2020–2023) across various hospitals. These cross-sectional, observational, and pilot designs demonstrate AI’s expanding use in healthcare and its potential to enhance clinical outcomes for diverse medical conditions, including chronic diseases (20). AI use has grown rapidly across Saudi Arabia’s academic, clinical, and public domains, with individuals increasingly relying on these platforms for health guidance. A local Makkah study revealed positive attitudes toward AI among adults with chronic diseases (21). Additional studies have documented high awareness of ChatGPT’s capabilities among graduate students, demonstrating its widespread accessibility and influence across different sectors (22–25).

Widespread adoption suggests many individuals, including parents seeking sensitive pediatric guidance, increasingly depending on public AI platforms. However, these “black box” systems risk generating inaccurate or inconsistent advice. Thus, evaluating AI’s pediatric diagnostic accuracy is clinically significant, as empirical evidence for differentiating ASD remains scarce. To address this gap, this study systematically evaluates the performance of AI platforms in identifying ASD from standardized pediatric case studies and distinguishing it from other neurodevelopmental conditions across three dimensions: diagnostic accuracy, concordance with clinical benchmarks, and ASD classification. By comparing multipurpose and medically oriented systems, this study highlights AI’s potential as a supportive tool for complex pediatric synthesis while investigating the risks of public reliance on potentially misleading automated information.

## 2 Materials and methods

### 2.1 Study design

In January 2026, a cross-sectional comparative in-silico case-based evaluation was carried out as an exploratory vignette benchmark to assess the baseline diagnostic-reasoning capabilities of AI platforms in analyzing and classifying pediatric clinical cases via standardized text-based prompts. The comparative analysis focused on evaluating general-purpose LLMs versus AI-assisted medical tools under standardized, controlled text-vignette conditions.

### 2.2 Study setting

The entire study was carried out remotely, without direct human participant involvement, utilizing online AI platforms. Both general-purpose models and available AI-assisted medical support tools were included in the platforms.

### 2.3 Study population (case dataset)

• ASD Group (n=10): diagnosed with Autism Spectrum Disorder (ASD)

• Non-ASD Group (n=10): diagnosed with ADHD, Speech & Language Delay, Selective Mutism, Depression, Childhood Anxiety Disorders

The inclusion of these specific non-ASD conditions was deliberate to challenge the AI’s ability to perform differential diagnosis among overlapping clinical presentations. Using peer-reviewed, well-documented cases in this *in-silico* study reduces variability and strengthens the assessment of the AI platforms’ interpretive quality.

### 2.4 Sampling and sample size

A purposeful sampling strategy was employed to select pediatric case studies containing clear, structured clinical information suitable for AI-based analysis. The primary objective was not to achieve population representativeness, but rather to provide well-defined, diagnostically rich cases that allow accurate evaluation of AI reasoning, diagnostic accuracy, and classification performance.

### 2.5 Data pre-processing and ethics

Data were sourced from published online clinical cases. To maintain integrity, narratives were kept verbatim, avoiding any paraphrasing or summarization, while all prior diagnoses were removed to ensure unbiased analysis. All personally identifiable information (PII) was redacted for anonymity, retaining only age and gender for diagnostic relevance. The comprehensive list of sources is maintained as a curated dataset available upon request.

### 2.6 AI platforms subjected to performance evaluation

Two categories of AI platforms were evaluated:

General-purpose Large Language Model Platforms (3):

• GPT-5.2 (OpenAI), Online, accessed: January 10–25, 2026

• Gemini 3 Pro (Google DeepMind), Online, accessed: January 10–25, 2026

• Claude Opus 4.5 (Anthropic), Online, accessed: January 10–25, 2026

AI-assisted medical support tools (3):

• Doctronic, (Online), accessed: January 10–25, 2026

• Personal Medical Analyst Jenova (Azeroth Inc.), (Online), accessed: January 10–25, 2026

• August a health AI (August AI), (Online), accessed: January 10–25, 2026

### 2.7 Case inclusion and exclusion criteria

Inclusion Criteria:

• Pediatric case studies published in peer-reviewed journals and documented clinical sources.

• Children aged 0–17 years

• Cases with confirmed diagnoses

Exclusion Criteria:

• Case studies of adults

• Cases requiring imaging, laboratory, or other non-textual assessments that AI cannot process

• Non-English publications without full translation

### 2.8 Study instrument (case standardization template)

A structured, standardized case-study template (Prompt) was developed to ensure consistency across AI evaluations. The Prompt included:

1. Patient Information: Age, gender, family background, school/grade level

2. Presenting Complaint/Observations: Parental, caregiver, or teacher observations regarding atypical behaviors

### 2.9 Prompt engineering strategy

This study employed a Zero-Shot Chain-of-Thought (CoT) framework. Unlike traditional prompting, this approach required models to articulate step-by-step logical deductions before reaching a diagnosis. The prompt was structured to mirror clinical workflows in two phases: Clinical Evidence Synthesis (deconstructing raw data) and Differential Mapping (cross-referencing with DSM-5 criteria). To ensure outputs relied solely on intrinsic knowledge, autonomous web-search was disabled, reflecting the models’ independent clinical reasoning capacity (26, 27).

### 2.10 Data collection procedure

• Each standardized case was submitted independently to every and each AI platforms separately.

• To preserve comparability control, a uniform prompt structure was used across platforms.

• The following AI outputs were methodically extracted:

1. Most likely diagnosis (with severity or classification: mild, moderate, severe)

2. Reasoning or justification

3. Confidence Level (estimated %),

• Each AI-generated response was recorded, coded, and stored in a secure database.

• Repeated trials (3 per case per platform) were performed to assess consistency and reliability. Each platform-case combination was analyzed through three different tests. The final classification was decided beforehand using majority vote from the three tests. Hence, each case represented one classification in the main analysis, whereas each output of the three tests was kept for analysis of consistency among platforms.

### 2.11 Data management

All AI-generated outputs were systematically coded and analyzed using Python, employing libraries for data manipulation, statistical computation, and visualization. Following data processing, all variables were reviewed for accuracy and completeness. Diagnostic accuracy was calculated as the proportion of AI-generated diagnoses that fully matched the reference diagnoses reported in the published case studies. Descriptive statistics, including frequencies and percentages, were produced for each AI platform to summarize diagnostic patterns and overall performance. To evaluate each platform’s ability to correctly identify ASD versus non-ASD pediatric conditions, sensitivity analyses and qualitative assessments of diagnostic reasoning were conducted. Concordance between AI-generated and reference diagnoses was examined using Cohen’s kappa, which provided a standardized measure of agreement beyond chance. Kappa values were interpreted using established benchmarks reflecting slight, fair, moderate, substantial, or near-perfect agreement. Percentage agreement was also calculated to complement kappa results and provide additional clarity regarding diagnostic consistency. Comparative analyses were then conducted to examine performance differences between general-purpose AI models and healthcare-specialized AI tools across accuracy, concordance, and classification metrics. Fisher’s exact test was applied to the aggregated 2×2 contingency data to evaluate potential differences in diagnostic accuracy between the two platform categories without violating statistical assumptions associated with small cell counts. Effect sizes were expressed using Odds Ratios (OR) with 95% Confidence Intervals (95% CI). All statistical tests were two-tailed, and a p-value (< 0.05) was considered statistically significant. All findings are presented in comprehensive tables and graphical summaries to support interpretation and enhance the visualization of results.

### 2.12 Ethical consideration

No human participants or personal data are involved. All case studies are sourced from publicly available, peer-reviewed publications.

## 3 Results

Ten pediatric cases diagnosed with Autism Spectrum Disorder (ASD) were included, comprising 7 males and 3 females, with ages ranging from 3 to 17 years (mean: 8.3 years). Most cases exhibited deficits in verbal or non-verbal communication, social withdrawal, repetitive behaviors, and sensory sensitivities. Severe behavioral manifestations, such as self-harm, selective mutism, and restricted eating, were reported in several cases. These standardized case studies were provided to all AI platforms for evaluation of diagnostic accuracy and classification performance (**Table 1**). Ten pediatric cases with non-ASD neurodevelopmental or psychiatric conditions were included, evenly split between males and females (5 each), with ages ranging from 4 to 17 years (mean: 11.1 years). Diagnoses included ADHD (4 cases), anxiety disorders (2 cases), selective mutism (2 cases), speech and language delay (1 case), and depression with stuttering (1 case). Frequently reported features included inattention, hyperactivity, social withdrawal, emotional dysregulation, and school refusal. These standardized cases were provided to all AI platforms to assess their ability to differentiate ASD from other overlapping pediatric conditions (**Table 2**).

**Table 1:** Baseline characteristics of the included pediatric cases studies (ASD).

Case N	Age	Gender	Diagnosis	Key clinical features/observations
1	6 years	M	ASD	No functional speech; poor receptive language; avoids eye contact; minimal response to name/instructions; hand-leading to communicate needs; social disengagement; limited sustained attention; not attending school due to speech limitation
2	6 years	F	ASD	Speech delay; social difficulties/no friends; restricted/repetitive play (lining up, resetting toys); rigidity/insistence on sameness; sensory sensitivity (noise/touch); meltdowns with self-/hetero-aggression; hyperactivity/inattention
3	3 years	M	ASD	No words by age 3; nonverbal communication mainly for regulation (hand-leading); limited eye gaze; inconsistent response to name; poor imitation; low tone/awkward motor skills
4	12 years	M	ASD	Limited verbal language; minimal interaction; unresponsive to name/directions; repetitive behaviors (pacing, hand flapping, humming, water play); feces smearing; sensory/behavioral dysregulation affecting hygiene/medical care
5	5 years	M	ASD	Touches genital and anal areas and engages in fecal-smearing on desks, walls, and other surfaces in classroom and public settings. Shows resistance to routine personal care, such as haircuts and dental visits, and no longer tolerates previously effective deep pressure, reflecting increased strength and oppositional behaviors.
6	13 years	M	ASD	Inconsistent school attendance with frequent early departures. Social withdrawal during breaks, avoidance of playground activities, and passive presentation without challenging behaviors. Marked anxiety, noise sensitivity, limited social/recreational engagement, and resistance to personal care (showering, tooth brushing).
7	9 years	M	ASD	Engages in self-harm (hand-biting, head-banging) and aggression toward others. Shows repetitive behaviors (hand-flapping, pacing, rocking, humming) and remains constantly active. Non-verbal, communicates via photographs, follows visual schedules, and transitions independently.
8	13 years	F	ASD	Selective mutism at school with refusal to participate or complete work. Communication is intact when calm but deteriorates under stress, with freezing, pacing, and noise sensitivity. Displays tactile sensitivities, postural difficulties, low energy, reduced eye contact, and preference for sedentary activities.
9	5 years	M	ASD	Non-verbal with limited non-verbal communication. Acute refusal of solid food for several days, despite a history of selective eating restricted to white or light-colored foods. Currently consuming only liquids, including juice and protein shakes.
10	17 years	F	ASD	Intense fixation on a fictional character with rigid, fixed ideas and prolonged engagement. Severe cognitive delay with minimal speech and disorganized reasoning. Avoidance of physical contact, limited eye contact, and behavior dominated by obsessions.

N, number; M, male; F, female.

**Table 2:** Baseline characteristics of the included pediatric cases studies (non-ASD).

Case N	Age	Gender	Diagnosis	Key clinical features/observations
11	9 years	M	ADHD, Combined Type	Impulsivity with social difficulties and unstable peer relationships. Academic disorganization, distractibility, poor instruction-following, frequent loss of belongings, sleep difficulties, resistance to routines, rushed hygiene, with motivation for academic success and friendships.
12	7 years	M	ADHD	Shows inattention, hyperactivity, impulsivity, emotional reactivity, defiance, and sleep difficulties, with socially immature and attention-seeking behaviors despite generally appropriate peer interactions.
13	13 years	F	ADHD	Shows inattention, distractibility, disorganization, impulsivity, mood fluctuations, anxiety, and difficulties with schoolwork, social interactions, and sleep routines, alongside low self-esteem and negative self-talk.
14	15 years	F	Anxiety	School avoidance, anxiety, panic attacks, low mood, irritability, sleep and appetite disturbances, worry about death, restlessness, and poor eye contact.
15	5 years	F	Separation Anxiety	Shows school refusal, separation anxiety, clinginess, nightmares, somatic complaints, regression in toileting, social withdrawal, and difficulty concentrating without maternal presence.
16	4 years	M	Speech Delay & language problem	Shows poor eye contact, limited communication and pre-linguistic skills, lack of social interaction, poor imitation, and difficulty engaging in play.
17	17 years	F	Selective Mutism	Shows persistent social silence, reliance on non-verbal gestures and nodding in school, school avoidance, and heightened morning anxiety, alongside compulsive checking behaviors, maintaining good academic performance, and effective eye contact.
18	10 years	F	ADHD	Shows poor academic performance, slow task completion, inattention, shyness, social withdrawal, and self-absorbed behaviors affecting school and social interactions.
19	13 years	M	Depression	Enuresis, mood instability, social withdrawal, bullying-related distress, poor emotional regulation, low affect, and suicidal ideation.
20	9 years	M	Separation anxiety	Shows school refusal, severe anxiety, panic-like behaviors, somatic complaints, social inhibition, phobias, and excessive worry about mother’s health.

N, number; M, male; F, female.

Among the 20 pediatric cases included in the study, 10 cases (50%) were diagnosed with ASD, while the remaining 10 cases (50%) were diagnosed with other pediatric conditions. Non-ASD diagnoses included ADHD (4 cases), anxiety disorders (2 cases), selective mutism (2 cases), speech and language delay (1 case), and depression (1 case) (**Figure 1**).

**Figure 1:**
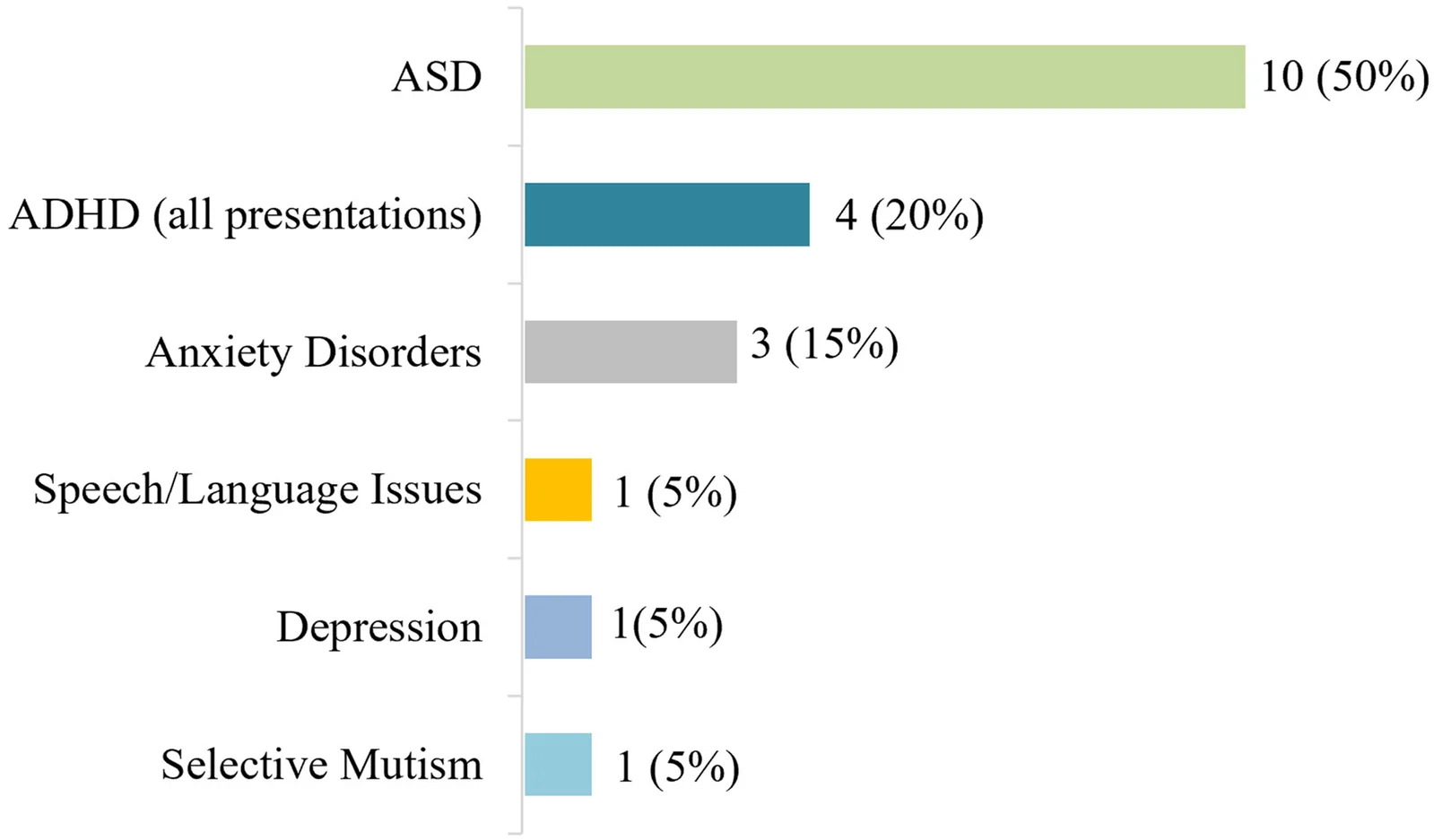
Distribution of diagnoses across study cases (N = 20).

Diagnostic accuracy among the six AI platforms ranged from 75% to 100%. Gemini 3 Pro led the performance with a flawless 100% correct classification, followed by Jenova AI at 95%. GPT-5.2, Claude Opus 4.5, and Doctronic each achieved 80% accuracy, while August AI had the lowest at 75% (**Table 3**).

**Table 3:** Performance metrics for AI diagnostic classification.

AI platform	Total cases	Correct diagnoses	Incorrect diagnoses	Accuracy (%) [95% CI]
GPT-5.2	20	16	4	(80) [56.3% – 94.3%]
Gemini 3 Pro	20	20	0	(100) [83.2% –100.0%]
Claude Opus 4.5	20	16	4	(80) [56.3% – 94.3%]
Doctronic	20	16	4	(80) [56.3% – 94.3%]
Jenova AI	20	19	1	(95) [75.1% – 99.9%]
August	20	15	5	(75) [50.9% – 91.3%]

True positives ranged from 6 to 10, with Gemini 3 Pro and Jenova AI achieving perfect detection (10), while GPT-5.2 recorded the lowest at 6. False negatives varied from 0 to 4. True negatives were highest at 10 for GPT-5.2 and Gemini 3 Pro, and lowest at 7 for August AI. False positives were minimal, with most platforms registering 0 or 1, except August AI with 3. Sensitivity ranged from 60% to 100%, and specificity from 70% to 100%. The table details AI classification outcomes in terms of correct and incorrect predictions based on the confusion-matrix framework (**Table 4**).

**Table 4:** Confusion-matrix summary of AI platforms’ performance in ASD classification.

AI platform	True positives (ASD)	False negatives	True negatives (Non-ASD)	False positives	Sensitivity (%) recall [95% CI]	Specificity (%) [95% CI]
GPT-5.2	6	4	10	0	(60) [26.2% – 87.8%]	(100) [69.2% –100%]
Gemini 3 Pro	10	0	10	0	(100) [69.2% – 100%]	(100) [69.2% – 100%]
Claude Opus 4.5	7	3	9	1	(70) [34.8% – 93.3%]	(90) [55.5% – 99.7%]
Doctronic	7	3	9	1	(70) [34.8% – 93.3%]	(90) [55.5% – 99.7%]
Jenova AI	10	0	9	1	(100) [69.2% – 100%]	(90) [55.5% – 99.7%]
August	7	3	7	3	(70) [34.8% – 93.3%]	(70) [34.8% – 93.3%]

Cohen’s Kappa values ranged from 0.56 to 0.859 across platforms. Gemini 3 Pro achieved the highest agreement (κ = 0.859; 90%), followed by Jenova AI (κ = 0.789; 85%). GPT-5.2 had the lowest agreement (κ = 0.56; 65%). Claude Opus 4.5, Doctronic, and August each demonstrated substantial agreement with κ values between 0.605 and 0.673 (70–75%) (**Table 5**).

**Table 5:** Platform-level multi-class agreement (Cohen’s Kappa).

Platform	Cohen’s Kappa (κ)	% Agreement	Interpretation
GPT-5.2	0.56	(65)	Moderate
Gemini 3 Pro	0.859	(90)	Mostly perfect
Claude Opus 4.5	0.617	(70)	Substantial
Doctronic	0.673	(75)	Substantial
Jenova AI	0.789	(85)	Mostly perfect
August	0.605	(70)	Substantial

Micro-averaged precision, recall, and F1 scores ranged from 0.65 to 0.9 across AI platforms. Gemini 3 Pro achieved the highest values in all three metrics (90%; F1 = 0.9), followed by Jenova AI (85%; F1 = 0.85). GPT-5.2 recorded the lowest scores (65%; F1 = 0.65), while Claude Opus 4.5, Doctronic, and August displayed intermediate performance between 70% and 75% (F1 = 0.7–0.75) (**Table 6**).

**Table 6:** Micro-averaged performance metrics of AI platforms.

AI group	Micro-precision	Micro-recall	Micro-F1
GPT-5.2	65	65	0.65
Gemini 3 Pro	90	90	0.9
Claude Opus 4.5	70	70	0.7
Doctronic	75	75	0.75
Jenova AI	85	85	0.85
August	70	70	0.7

A Fisher’s Exact Test was also conducted for enhanced clarity. General-purpose AI platforms produced 52 correct and 8 incorrect classifications (86.7%), whereas healthcare-assisted AI platforms produced 50 correct and 10 incorrect classifications (83.3%). Fisher’s Exact Test demonstrated no statistically significant difference between the two categories (two-sided p = 0.799) (**Table 7**).

**Table 7:** Fisher’s exact test comparing diagnostic accuracy across AI platform categories.

Variable	General-purpose AI	AI-assisted medical	Fisher’s value	p-value
Correct Classifications	52	50	1.30	> 0.799
Incorrect Classifications	8	10		

Gemini 3 Pro demonstrated the fastest thought time (0.525 s), while August was the slowest (13.07 s). For full response time, Doctronic was the quickest (4.46 s), whereas Claude Opus 4.5 was the slowest (370.17 s, 6.10 min) (**Table 8**).

**Table 8:** Latency and response performance of AI platforms.

Platform	Mean latency (Thought Time) (s)	Benchmark classification	Mean full response time (s)	Benchmark classification
GPT-5.2	*10.17s*	Slow	*39.17s*	Slow
Gemini 3 Pro	*0.525s*	Fast	*31.16s*	Slow
Claude Opus 4.5	*4.71 s*	Moderate	*370.17s = 6.10 min*	Very Slow
Doctronic	3.19s	Moderate	*4.46s*	Excellent
Jenova AI	12.24s	Slow	*19.02s*	Acceptable
August	13.07s	Slow	*20.41s*	Acceptable

S, seconds.

Gemini 3 Pro ranked first in diagnostic accuracy, followed by Jenova AI in second place. GPT-5.2, Claude Opus 4.5, and Doctronic shared the third position (**Figure 2**).

**Figure 2:**
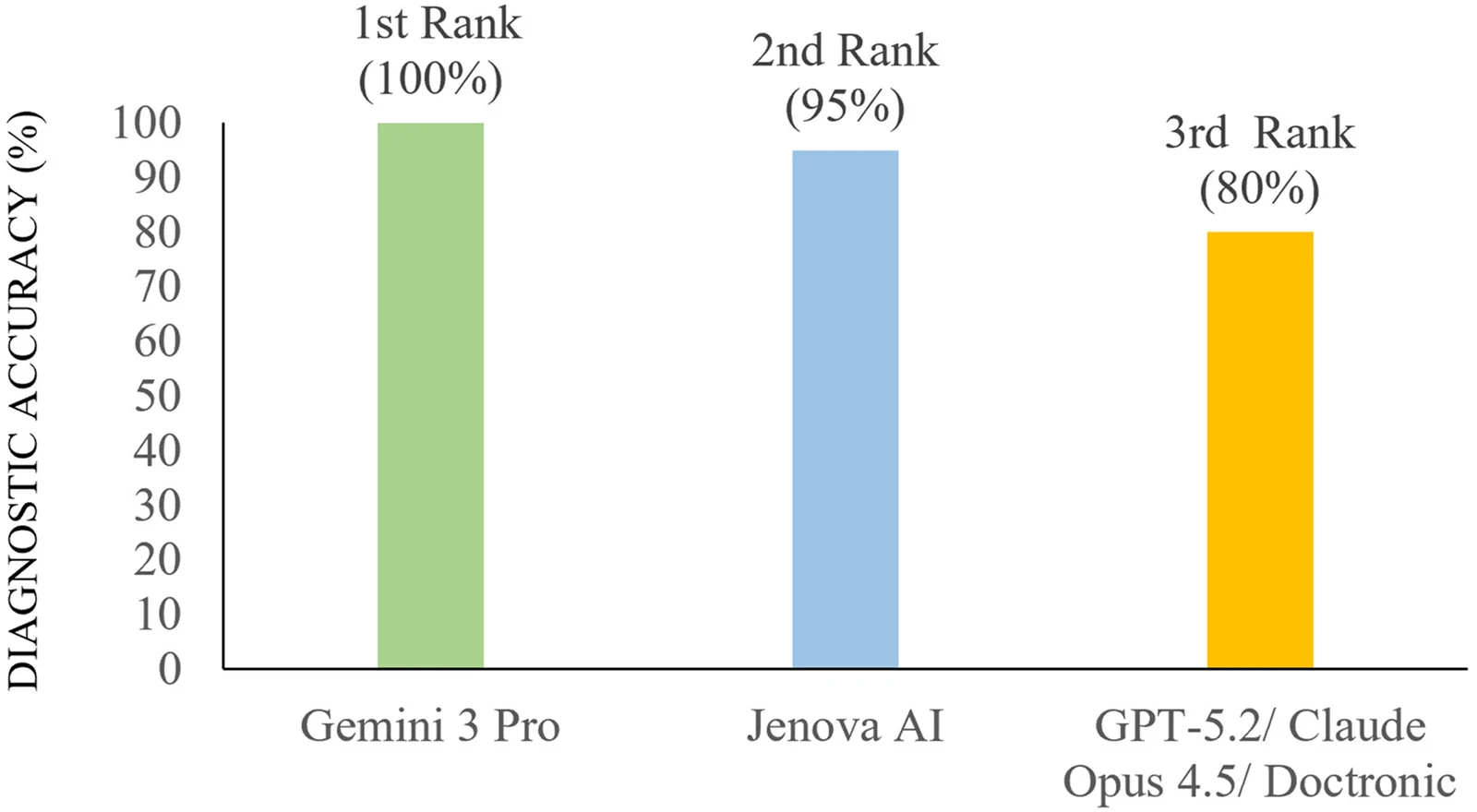
Ranking of the top three AI platforms according to diagnostic accuracy.

All platforms fully adhered to the specified prompts for providing the primary probabilistic diagnosis, severity assessment, diagnostic rationale, and confidence levels, with no deviations observed. Among all platforms, Claude and Jenova demonstrated the highest extrapolative capabilities, with Claude outperforming Jenova by producing additional extrapolative outputs, including clinical recommendations, urgent recommendations, co-occurring conditions, predictions, and analysis of challenging behaviors (**Table 9**).

**Table 9:** Cross-platform adherence to prompts and extrapolative output capabilities.

Evaluation category	Output feature	GPT	Gemini	Claude	Doctronic	Jenova	August
Prompt-Adherent Outputs	Primary Probabilistic Diagnosis	Yes	Yes	Yes	Yes	Yes	Yes
	Severity Assessment	Yes	Yes	Yes	Yes	Yes	Yes
	Diagnostic Rationale	Yes	Yes	Yes	Yes	Yes	Yes
	Confidence Level	Yes	Yes	Yes	Yes	Yes	Yes
Extrapolative Outputs	++Clinical Recommendations	No	No	Yes	No	Yes	No
	++Urgent Clinical Recommendations	No	No	Yes	No	No	No
	++Co-occurring Conditions to Consider	No	No	Yes	No	No	No
	++ Predictions and Support Needs	No	No	Yes	No	Yes	No
	++Analysis of Challenging Behaviors	No	No	Yes	No	No	No

## 4 Discussion

The findings of this study offer a critical vantage point into the evolving landscape of AI-driven diagnostics, specifically regarding the comparative efficacy of general-purpose Large Language Models (LLMs) and specialized medical AI platforms in identifying Autism Spectrum Disorder (ASD). The results in this study unveil a compelling paradox: while specialized platforms are architecturally designed for clinical precision, general-purpose models demonstrated an ability to navigate the nuances of complex differential diagnosis. Consistent with 2025 systematic reviews highlighting the potential of machine learning in processing multi-modal diagnostic data (28), this study confirms that this trend extends to textual clinical interpretation. Furthermore, this study is consistent with a recent study (29), that showed that generative language learning models outperform specialized classifiers in accurately identifying the precise indicators associated with autism spectrum disorder.

The diagnostic performance of the AI platforms in this study, particularly Gemini 3 Pro and Jenova, demonstrates a significant advancement when compared to contemporary benchmarks in the literature. Recent meta-analyses from 2025 report (28, 30), that conventional Machine Learning (ML) and Deep Learning (DL) models typically achieve sensitivities of 91.6% and 87.3% respectively. In contrast, the findings of this study reveal that both Gemini 3 Pro and Jenova achieved a superior sensitivity of 100%. This suggests that large language models (LLMs) may possess a more robust capacity for “rule-out” diagnostics in neurodevelopmental cases than traditional algorithmic approaches.

A fundamental strength identified in LLMs is their superior proficiency in synthesizing and interpreting unstructured data formats. Unlike traditional algorithms, LLMs excel in parsing open-ended textual descriptions, conversational narratives, and complex dialogue-based interactions (31, 32). This linguistic flexibility is particularly vital in the field of ASD, where diagnostic clarity often hinges on qualitative, narrative-rich details that describe behavioral nuances and social-communication patterns. According to published studies, kappa values obtained from strong to near-complete agreement between evaluators indicate this. This is consistent with the results of this study, where kappa values ranged from strong to near-complete agreement between evaluators (30).

Furthermore, the performance profiles observed in other platforms, such as GPT-5.2 (Sensitivity 60%) and August (Sensitivity 70%), fall within the varying performance bands documented in recent clinical AI reviews. This distinction suggests that general-purpose LLMs possess diverse architectural strengths, which influence their approach to complex clinical interpretation. While some platforms demonstrate high recall suitable for screening assistance, others exhibit complementary characteristics. Rather than indicating a universal standard, these findings highlight that different models are optimized for different clinical tasks, reflecting the heterogeneous nature of current generative AI applications as identified in recent systematic reviews.

Furthermore, these findings offer a significant point of departure from earlier exploratory studies concerning ChatGPT and its ability to identify early signs of Autism Spectrum Disorder (ASD). Previous research primarily established a “proof of concept” by inducing models like GPT-3.5-Turbo and GPT-4 to generate approximately 4,200 artificial text snippets representing hypothetical child behaviors. While those studies reported an 83% accuracy rate in aligning these self-generated snippets with DSM-5 criteria, this study reveals a different reality when these models are tasked with independent clinical interpretation.

Furthermore, these findings offer a valuable point of comparison to earlier exploratory studies concerning ChatGPT’s capability in identifying early signs of Autism Spectrum Disorder (ASD). Previous research established an important ‘proof of concept’ by utilizing models like GPT-3.5-Turbo and GPT-4 to generate hypothetical clinical descriptions, reporting an 83% accuracy rate in aligning these self-generated snippets with DSM-5 criteria (33, 34)]. However, evaluating these models on independent clinical cases presents distinct performance dynamics. Specifically, in contrast to the high performance reported in self-generated contexts, this study observed that ChatGPT (GPT-4o) demonstrated different sensitivity and accuracy profiles when evaluated against standardized, real-world textual case studies. While prior work highlighted an 83% accuracy within structured generative environments, our simulation suggests that interpretive performance varies depending on the evaluation framework, highlighting the diverse operational profiles and distinct strengths of different AI platforms, such as Gemini 3 Pro.

This distinction is important, as it suggests that while ChatGPT maintains consistency in structuring and generating symptomatic descriptions, its performance can vary when interpreting complex, multi-layered clinical narratives. Consequently, the 83% accuracy observed in prior preliminary studies may reflect performance under more structured conditions, whereas clinical utility in nuanced cases presents further areas for development. These findings highlight the value of distinguishing between the model’s generative capacity and its interpretive diagnostic application. The analysis in this study further aligns with contemporary literature regarding the functional role of AI in supporting caregivers. Recent studies, such as (35), indicate that models like ChatGPT possess the potential to offer parents and caregivers accurate, empathetic, and highly accessible information about Autism, acting as a vital complement to professional guidance.

Consequently, this validates the ongoing necessity for rigorous supervision and continuous development. The “information gap” identified in this research suggests that while AI can serve as an accessible first-line resource for families, it must be developed within the stratified framework this study proposes—ensuring that information provided to caregivers is strictly validated and focused, thereby preventing the risks associated with the misinterpretation of ASD signs.

In contrast to the findings of the present study, earlier comparative benchmarks—such as the OpenAI reports—positioned Gemini 2.5 Pro Preview among the lower-performing models in complex reasoning tasks, with a reported accuracy of 52.1% ± 1.4%, compared to OpenAI’s (67.8%) and GPT (63.9%) (34). In the current evaluation, Gemini 3 Pro demonstrated markedly improved sensitivity and specificity in the interpretation of ASD-related clinical narratives. This divergence may reflect ongoing model refinement and maturation from preview-stage implementations to more stable iterations, rather than a definitive indication of architectural superiority. More broadly, these findings underscore the dynamic nature of the AI landscape, in which model performance rankings may shift across development cycles. Nevertheless, the results should be interpreted cautiously given the limited sample size and the reliance on previously published case materials rather than supervised clinical data. Accordingly, the observed performance gains suggest potential methodological advancement.

Notably, the data revealed that no AI platform experienced a total failure; all systems demonstrated a baseline capacity to recognize neurodevelopmental conditions, with none scoring zero across the evaluated cases. This finding suggests that contemporary AI architectures—regardless of specialization—possess a foundational level of competency in neurodevelopmental semiology when provided with structured clinical information. This observation is consistent with the findings of current study in 2026 (30, 36), which indicate that advanced large language models have reached a degree of inferential maturity enabling accurate processing of complex behavioral and developmental markers. Within this overall pattern of baseline competency, Gemini 3 Pro emerged as the highest-performing system in the present study. Despite its general-purpose design, Gemini demonstrated consistently accurate classification of ASD and related differential diagnoses, alongside efficient response latency and strong adherence to the provided clinical prompts. Rather than reflecting speed or output verbosity alone, this performance suggests effective integration of reasoning depth and task-focused response generation.

Gemini’s performance is notable, as it surpassed specialized platforms like Jenova—which recorded a score of 19/20 with a longer latency—and exhibited higher diagnostic precision than other general-purpose models. While some AI systems emphasize conversational summaries, Gemini’s success suggests that neurodevelopmental diagnostics can benefit from “reasoning-heavy” frameworks where speed, accuracy, and clinical rigor converge. These results contrast with literature suggesting that general-purpose models underperform compared to ASD-specific tools (37). This divergence indicates that the multimodal training of advanced LLMs may provide more “reasoning fluidity” than the structured constraints of specialized systems, overcoming what this study identifies as the “specialization bottleneck”.

Furthermore, our aggregated statistical comparison between general-purpose and healthcare-specialized AI models yielded no statistically significant variation in performance (p = > 0.799). This suggests that general-purpose foundation models, when provided with structured clinical prompts, can perform at a baseline classification accuracy comparable to platforms tailored for healthcare context. While tools marketed for healthcare applications often emphasize domain-specific terminology alignment, general-purpose models demonstrated similar proficiency in categorizing standardized narrative cases within this sample.

### 4.1 Strengths and limitations

This study’s primary strength is its evaluation of next-generation LLMs (e.g., Gemini 3 Pro and Claude Opus) within the 2025–2026 landscape. Utilizing narrative-rich clinical case studies rather than synthetic snippets allowed for a practical assessment of diagnostic reasoning. Furthermore, the cross-platform comparison offered useful insights into the comparative performance between general-purpose models and specialized medical platforms. Conversely, the modest sample size (n=20; 10 ASD and 10 non-ASD cases) constrained statistical power for subgroup comparisons and generalizability. Consequently, diagnostic performance metrics are interpreted as exploratory baseline estimates, accompanied by corresponding 95% confidence intervals. Additionally, the reliance on textual narratives omitted visual and behavioral observations critical to clinical ASD evaluation. Given the rapid evolution of AI architectures, these results represent a temporal snapshot; performance may vary as model versions and prompting strategies evolve. Therefore, future prospective studies utilizing larger, multi-center datasets are needed to confirm these exploratory findings and evaluate real-world clinical utility. Additionally, the study relied on heterogeneous case sources from published literature without a single uniform, independent diagnostic gold standard applied across all cases, which may introduce variability in narrative detail and diagnostic criteria.

### 4.2 The pedagogical-clinical tier (educational AI)

The creative and exhaustive nature of platforms like Claude identifies a clear functional niche for medical education. This study suggests that specialized “Educational AI” models be optimized for medical students and trainees. Unlike rapid diagnostic engines, these systems should be designed to prioritize creative clinical synthesis and in-depth differential mapping. By providing exhaustive rationales and opening new conceptual horizons, such models would function as “pedagogical companions,” fostering critical thinking in a controlled, simulated learning environment.

### 4.3 The community and patient-centric tier (public health AI)

To address the needs of patients and their families, this study proposes a dedicated “Public Health AI” tier. This model would be engineered for accessibility, ensuring it respects diverse cultural, age-related, and educational backgrounds. To mitigate the risk of misinterpretation, the study recommends that this tier remains purposefully restricted and focused, avoiding creative extrapolation. Its primary utility would be to provide simplified guidance for low-acuity, non-urgent inquiries. By facilitating home-care procedures for minor cases, this system could strategically reduce the burden on emergency facilities, ensuring priority for urgent clinical cases without substituting the essential role of the physician.

### 4.4 The professional advanced tier (clinical decision support)

For healthcare professionals, the results advocate for an “Advanced Clinical AI”—a high-speed, high-precision engine modeled after the efficiency observed in Gemini. This study suggests that access to such sophisticated systems be strictly regulated via authorized organizational credentials (User ID/Password). This ensures that high-level diagnostic capabilities are reserved for certified practitioners, allowing for rapid, evidence-based intervention support within a secure and professional framework.

Optimizing generative AI outputs is crucial in ASD diagnostics, where the management of complex or ambiguous cases requires high precision (38, 39). To enhance the clinical utility and reliability of these tools, implementing standardized input protocols and structured operational frameworks is highly beneficial. Incorporating real-time instructional guidance and establishing robust guardrails into health-oriented AI interfaces can further support this process. Such measures facilitate a supervised ‘human-in-the-loop’ approach, effectively leveraging the analytical capabilities of AI while maintaining rigorous standards of clinical accuracy.

## 5 Conclusion

In this exploratory in-silico benchmark, the ability of the tested AI platforms to classify pediatric clinical vignettes in a standard format was shown, with different degrees of agreement with reference classifications. Gemini 3 Pro and Jenova AI demonstrated the best overall performance. Additionally, models such as Claude Opus 4.5 exhibited valuable reasoning flexibility in interpreting complex Autism Spectrum Disorder (ASD) presentations, highlighting their potential role as supportive tools for narrative analysis and medical education. However, given the small sample size and vignette-based methodology, these estimates remain preliminary, providing comparative insights rather than direct evidence of clinical utility. This study did not show the validity of using these platforms for ASD screening or diagnosis, nor does it indicate their use in the clinical diagnostic process. Moving forward, integrating AI into autism care requires standardized input protocols and a tiered framework tailored for families, trainees, and specialists, ensuring that while AI cannot replace human clinical judgment, it can serve as a supportive tool for narrative analysis and case interpretation.

## Data Availability

The raw data supporting the conclusions of this article will be made available by the authors, without undue reservation.
