# Supplementary material for: An in-silico comparative cross-sectional diagnostic- accuracy study evaluating artificial intelligence platforms in identifying autism spectrum disorder from standardized pediatric case vignettes 2026
Source: Front Psychiatry. 2026 Sep 15;17:1941175. doi: 10.3389/fpsyt.2026.1941175 (PMC13619823; doi:10.3389/fpsyt.2026.1941175)
Supplement: Supplementary file 1 [file DataSheet1.pdf]

Published Sources of the 20 Pediatric Case Studies Used as Input Cases

Case	Diagnosis	Study Title	Source / DOI
1	ASD	A CASE STUDY OF AUTISM SPECTRUM DISORDER IN 6 YEAR'S OLD BOY: ASSESSMENT AND DIAGNOSIS	10.5281/zenodo.16355731
2	ASD	Clinical case-Autism spectrum disorder	10.15406/jmen.2023.11.00403
3	ASD	Children with Autism Spectrum Disorders	10.1044/leader.FTR2.15012010.1
4	ASD	Middletown Autism Center. *Case study 1*	https://sensory-processing.middletownautism.com/casestudies/case-study-1/
5	ASD	Middletown Autism Center. *Case study 2*	https://sensory-processing.middletownautism.com/casestudies/case-study-2/
6	ASD	Middletown Autism Center. *Case study 3*	https://sensory-processing.middletownautism.com/casestudies/case-study-3/
7	ASD	Middletown Autism Center. *Case study 4*	https://sensory-processing.middletownautism.com/casestudies/case-study-4/
8	ASD	Middletown Autism Center. *Case study 6*	https://sensory-processing.middletownautism.com/casestudies/case-study-6/
9	ASD	ASD-CS (CDM) - Comprehensive Case Study of Autism Spectrum Disorder – Studocu	https://www.studocu.com/en-us/document/keiser-university/care-management/asd-cs-cdm-case-study/112853686
10	ASD	Autistic spectrum disorder and fairy tales: A case study	10.5348/101414Z01ES2023CR
11	ADHD-C	Pediatric Case Study: Child with ADHD	https://www.occupationaltherapy.com/articles/pediatric-case-study-child-with-5145-5145
12	ADHD-C	The College of Family Physicians of Canada Case Study 1 - Jack	https://fmf.cfpc.ca/wp-content/uploads/2016/11/S133451_Best-Practices-for-ADHD-Across-the-Lifespan.pdf
13	ADHD	The College of Family Physicians of Canada Case Study 2 - Nancy	https://fmf.cfpc.ca/wp-content/uploads/2016/11/S133451_Best-Practices-for-ADHD-Across-the-Lifespan.pdf
14	Anxiety	Treating anxiety in pediatric primary care: A case report	10.1016/j.pedn.2025.02.012
15	Separation Anxiety	Separation Anxiety Disorder (SAD) in a 5-year-old Child: A Case Report	10.2991/978-94-6463-310-8_23
16	Selective Mutism	17-year-old Selective Mutism Case without Treatment for A Long Time	10.18678/dtfd.1092388
17	Speech Delay	Speech Delay in Early Childhood: A Case Study of a 3.5-Year-Old Child	10.24256/ideas.v12i2.5809
18	ADHD-I	A Case Study in Attention-Deficit/Hyperactivity Disorder: An Innovative Neurofeedback-Based Approach	10.3390/ijerph19010191
19	Depression	Case Study of a 13-year-old Boy Suffering from Depression and Stuttering	https://www.researchgate.net/publication/338717794_Case_Study_of_a_13-year-old_Boy_Suffering_from_Depression_and_Stuttering
20	Separation anxiety	Separation anxiety disorder and school refusal in children and adolescents	10.1542/pir.27-2-56
