# Supplementary material for: An in-silico comparative cross-sectional diagnostic- accuracy study evaluating artificial intelligence platforms in identifying autism spectrum disorder from standardized pediatric case vignettes 2026
Source: Front Psychiatry. 2026 Sep 15;17:1941175. doi: 10.3389/fpsyt.2026.1941175 (PMC13619823; doi:10.3389/fpsyt.2026.1941175)
Supplement: Supplementary file 2 [file DataSheet2.pdf]

Supplementary Material

Structured Prompt Components (Tabular Summary)

Section	Prompt Content
**Instruction**	Objective: Perform a systematic clinical analysis of the provided pediatric case study to evaluate diagnostic alignment with the DSM-5 framework using **internal probabilistic reasoning.**<br>Analytical Workflow:<br>1. Deconstruction: Identify all clinical markers from the provided case.<br>2. Differential Mapping: Cross-reference these markers against DSM-5 criteria using internal knowledge only. (Web search is strictly prohibited).<br>3. Synthesis: Derive the most likely clinical conclusion based on the available data.<br>Constraints:<br>▪ Use **only the information provided** in the case.<br>▪ Do **not provide a definitive clinical diagnosis**; this is for research/educational purposes.<br>▪ Present the response in a clear, structured format using numbered lists.<br>**Do not use any external sources or search online. Use only your reasoning as a clinical analyst
**Requested Output**	1. Most likely diagnosis (severity/classification level: mild/moderate/severe)<br>2. Reasoning / justification<br>3. Confidence Level
**Case Input**	Patient Information, Presenting Complaint / Observations
